# Synthesis and Characterization of Green ZnO@polynaniline/Bentonite Tripartite Structure (G.Zn@PN/BE) as Adsorbent for As (V) Ions: Integration, Steric, and Energetic Properties

**DOI:** 10.3390/polym14122329

**Published:** 2022-06-09

**Authors:** Mohamed Abdel Salam, Mohamed Mokhtar, Soha M. Albukhari, Doaa F. Baamer, Leonardo Palmisano, Mariusz Jaremko, Mostafa R. Abukhadra

**Affiliations:** 1 Department of Chemistry, Faculty of Science, King Abdulaziz University, P.O. Box 80200, Jeddah 21589, Saudi Arabia; mabdelsalam@kau.edu.sa (M.A.S.); mmoustafa@kau.edu.sa (M.M.); salbukhari@kau.edu.sa (S.M.A.); dfbaamer@kau.edu.sa (D.F.B.); 2 Schiavello-Grillone Photocatalysis Group, Dipartimento di Ingegneria, Università degli Studi di Palermo, Viale delle Scienze (Ed. 6), 90128 Palermo, Italy; leonardo.palmisano@unuipa.it; 3 Smart-Health Initiative (SHI), Red Sea Research Center (RSRC), Biological and Environmental Science and Engineering (BESE) Division, King Abdullah University of Science and Technology (KAUST), P.O. Box 4700, Thuwal 23955-6900, Saudi Arabia; mariusz.jaremko@kaust.edu.sa; 4 Geology Department, Faculty of Science, Beni-Suef University, Beni-Suef City 62511, Egypt; 5 Materials Technologies and Their Applications Lab, Geology Department, Faculty of Science, Beni-Suef University, Beni-Suef City 62111, Egypt

**Keywords:** bentonite, polynailine, ZnO, As (V), adsorption, equilibrium models

## Abstract

A green ZnO@polynaniline/bentonite composite (G.Zn@PN/BE) was synthesized as an enhanced adsorbent for As (V) ions. Its adsorption properties were assessed in comparison with the integrated components of bentonite (BE) and polyaniline/bentonite (PN/BE) composites. The G.Zn@PN/BE composite achieved an As (V) retention capacity (213 mg/g) higher than BE (72.7 mg/g) and PN/BE (119.8 mg/g). The enhanced capacity of G.Zn@PN/BE was studied using classic (Langmuir) and advanced equilibrium (monolayer model of one energy) models. Considering the steric properties, the structure of G.Zn@PN/BE demonstrated a higher density of active sites (Nm = 109.8 (20 °C), 108.9 (30 °C), and 67.8 mg/g (40 °C)) than BE and PN/BE. This declared the effect of the integration process in inducing the retention capacity by increasing the quantities of the active sites. The number of adsorbed As (V) ions per site (1.76 up to 2.13) signifies the retention of two or three ions per site by a multi-ionic mechanism. The adsorption energies (from −3.07 to −3.26 kJ/mol) suggested physical retention mechanisms (hydrogen bonding and dipole bonding forces). The adsorption energy, internal energy, and free enthalpy reflected the exothermic, feasible, and spontaneous nature of the retention process. The structure is of significant As (V) uptake capacity in the existence of competitive anions or metal ions.

## 1. Introduction

The continuous increase in the levels of toxic heavy metal ions in major water supplies and freshwater resources as a result of extensive metallurgical, industrial, and mining activities represents critical challenges for the safe future of humanity [1,2]. Such metals have high accumulation and toxicity behavior, which classifies them as hazardous pollutants for humans, animals, and aquatic organisms [3,4]. Dissolved arsenic ions (As) have been recognized as highly toxic forms of water contaminants [5,6]. The recommended level of As (V) in water resources should be less than 10 µg/L, beyond this value it will be of significant carcinogenic effects on kidney, skin, lung, and urinary bladder [5,7]. Moreover, several diseases such as esophageal, cardiovascular disease, abdominal pain, bloody diarrhea, vomiting, and diabetes have been documented as the results of the impact of As (V) pollutants [8,9].

Several techniques have been suggested to reduce As (V) concentrations to acceptable levels, such as chemical precipitation, membrane filtration, ion exchange purification, biological remediation, and adsorption [10,11]. The decontamination of As (V) by adsorption is recommended in several studies as a low-cost, simple, recyclable method [8]. Co-Zn-ZIF [12], ZnO/zeolite [13], MWCNTs [14], iron oxyhydroxide [10], Fe/biochar [7], activated carbon [15] and goethite [16] were studied recently as promising adsorbents for As (V) ions from water. The selection of suitable adsorbents depends on the production cost, the fabrication simplicity, adsorption kinetics, and adsorption capacity.

Recently, the heterogeneous and multifunctional synthetic structures of organic and inorganic components were investigated as very effective adsorbents for both metal ions and dissolved organic chemicals [17,18]. The clay-based hybrid structures with organic polymers and metal oxides, especially bentonite-based composites, have been introduced in more recent years as the best multifunction structures for the retention of toxic metal ions from water supplies [19,20]. Bentonite is a highly available smectite-bearing natural material and has significant adsorption properties, either as a single-phase or as an integrated component in composite [21]. Technically, it has significant surface reactivity, ion exchange properties, flexible structure, adsorption capacity, nontoxicity, biocompatibility, and surface area [5,22].

Recently, several studies demonstrated the valuable impact of the integration processes between the bentonite layers and different species of polymers on the physicochemical properties of the structure as an adsorbent [17,22,23]. Among the addressed polymers, polyaniline is a highly recommended polymer that has inexpensive and facile production procedures, in addition to a high surface area, safety, adsorption capacity, and oxidation properties [24,25]. It was reported that the intercalation of the bentonite layers with the polyaniline chains resulted in an advanced product of enhanced surface area, conductivity, mechanical stability, and adsorption capacity [25,26,27].

Additionally, the integration between bentonite and some metal oxides or metal-based structures was reported as an effective technique to induce the physicochemical properties of bentonite. This included nickel Fe/Mg LDH [21], magnesium ferrite [28], MoS_2_ [29], MgFe_2_O_4_ [30], ZnO/CuO [31], and Bi_2_O_3_-ZnO [22]. ZnO nanoparticles, in addition to their base materials, have been assessed extensively as effective adsorbents and photocatalysts in the literature [32,33]. ZnO-based structures have a high surface area, stability, non-toxicity, adsorption capacity, and photocatalytic activity [32,34,35]. Several fabrication methods were applied during the synthesis of ZnO as co-precipitation, hydrothermal, sol–gel, and green methods [36,37]. The green methods are environmental techniques that involve the application of the liquid extracts of algae, plants, and leaves as capping and reducing reagents during the precipitation of the metals and metal oxides [36]. Moreover, this method resulted in non-agglomerated nanoparticles using an inexpensive technique and low quantities of toxic chemicals [38,39]. The previous studies reported that the existence of ZnO as a component in different composites or supporting it into suitable carrier resulted in innovative hybrid structures with enhanced adsorption and recovery properties [32,36].

Based on our previous study, the synthesis of a tripartite hybrid structure from bentonite, polyaniline, and green ZnO will result in new multifunctional material with enhanced physicochemical properties, which qualify it to be applied as an enhanced adsorbent material [37]. Therefore, the presented study involved a detailed investigation of the adsorption properties of the green ZnO@polyaniline/bentonite composite (G.Zn@PN/BE) as an enhanced adsorbent for As (V) ions. This involved an assessment of the essential variables, including the effect of the integrated components. The influence of the integrated component and the main As (V) uptake mechanism was illustrated based on normal equilibrium studies and advanced equilibrium studies based on the statistical physics theory, considering the energetic and steric parameters.

## 2. Experimental Work

### 2.1. Materials

Natural bentonite was delivered directly from a bentonite quarry south of El Hammam city in the Northern Western Desert, Egypt. Aniline monomer, dimethylsulfoxide, and (NH_4_)_2_S_2_O_8_ (Winlab company; UK.) were used during the preparation of the polyaniline polymer. Commercial leaves of green tea were used to prepare the oxidizing and capping extract and zinc nitrate hexahydrate powder (Zn(NO_3_)_2_.6H_2_O) (Sigma-Aldrich, St. Louis, MI, USA) were applied during the green production of ZnO. Diluted nitric acid (0.1 M) and diluted NaOH solution (0.1 M) were used during the adjustment of the pH value.

### 2.2. Synthesis of Green ZnO@polyaniline/Bentonite Composite (G.Zn@PN/BE)

The intercalation of the bentonite layers with polyaniline occurred by direct polymerization of the monomer (aniline) in the presence of bentonite suspension. The typical procedures involved dissolving of the monomer (0.1 M) in HCl solution (0.5 M) followed by the addition of (NH_4_)_2_S_2_O_8_ solution (0.15 M). During the initial precipitation of polyaniline (PN), the bentonite (BE) suspension (1 g within 50 mL of water) was added to it under adjustable stirring for 24 h. After this period, the PN/BE composite was filtrated, washed, and dried for 24 h under room conditions. After that, the prepared PN/BE particles were homogenized within 100 mL of an aqueous solution of zinc nitrate (0.4 M) by stirring (500 rpm) and under the effect of ultrasonic waves (240 W). The liquid extract of green tea was prepared according to Salam et al., [38] and added to the aqueous solution of zinc in the presence of PN/BE particles. After the homogenization period of 120 min, the reactants were left in the open environment for 24 h to ensure the combination of the formed nano ZnO and the dispersed PN/BE particles. Finally, the separated G.Zn@PN/BE fractions were dried gently for 12 h at 65 °C to be assessed in further studies. All the selected ratios of the component were selected after a series of primary tests.

### 2.3. Analytical Techniques

The crystalline phases and structures were assessed based on the XRD peaks of the materials using an X-ray diffractometer (PANalytical-Empyrean type; Eindhoven, The Netherland). The morphological forms on the investigated components and their composites were assessed depending on their SEM images (Gemini, New York, NY, USA; Zeiss-Ultra 55 Scanning-Electron Microscope) and HRTEM images (JEOL Ltd., Tokyo, Japan; JEOL-JEM, 2100Transmission Electron Microscope). The functional groups of the integrated materials and their composites were studied considering their FT-IR spectra, utilizing a Fourier Transform Infrared spectrometer (FTIR−8400S). The microstructural properties of the surface area and porosity were determined by the Beckman Coulter ((Brea, CA, USA)) surface area analyzer (SA3100 type) considering the N_2_ adsorption/desorption curve.

### 2.4. Adsorption Studies

The adsorption tests of As (V) were designed as batch experiments including the effect of pH (2 until 8), contact time (5–840 min), G.Zn@PN/BE dosages (0.1–0.6 g/L), As (V) concentration (50–350 mg/L) and temperature (20–40 °C) at a certain volume (200 mL). The experiments were repeated for three runs considering the average values of the results with standard deviations < 5.2%. The treated solutions were acidified with nitric acid (2%) and the remaining As (V) concentrations were measured by inductively-coupled-plasma mass spectrometry (Perkin Elmer). The As (V) adsorption capacity (*Q_e_*) was calculated from Equation (1).

(1) $$Q_{e\;\left(\mathrm{mg}/g\right)}=\frac{(C_{o}-C_{e})V}{m}$$

The assessed kinetic and classic equilibrium models (Table S1) were inspected considering the fitting parameters of the correlation coefficient (R^2^) (Equation (2)) and Chi-squared (χ^2^) (Equation (3)).

(2) $$R^{2}=1-\frac{\sum {\left(Q_{e,\;exp}-Q_{e,\;cal}\right)}^{2}}{\sum {\left(Q_{e,\;exp}-Q_{e,\;mean}\right)}^{2}}$$

(3) $$\chi^{2}=\sum \frac{{\left(Q_{e,\;exp}-Q_{e,\;cal}\right)}^{2}}{Q_{e,\;cal}}$$

The fitting degrees with the advanced equilibrium models (Table S1) were assessed depending on both the correlation coefficient (R^2^) and root mean square error (*RMSE*) (Equation (4)).

(4) $$RMSE=\sqrt{\frac{\sum_{i=1}^{m}{\left(Qi_{cal}-Qi_{exp}\right)}^{2}}{m^{\prime }-p}}$$

The *m*′, *p*, *Qi_cal_*, and *Qi_exp_* symbols denote the inserted experimental data, number of studied variables, As (V) adsorbed quantities, and actual As (V) adsorbed quantities, respectively.

## 3. Results and Discussion

### 3.1. Characterization of G.Zn@PN/BE Structure

**Figure 1:**
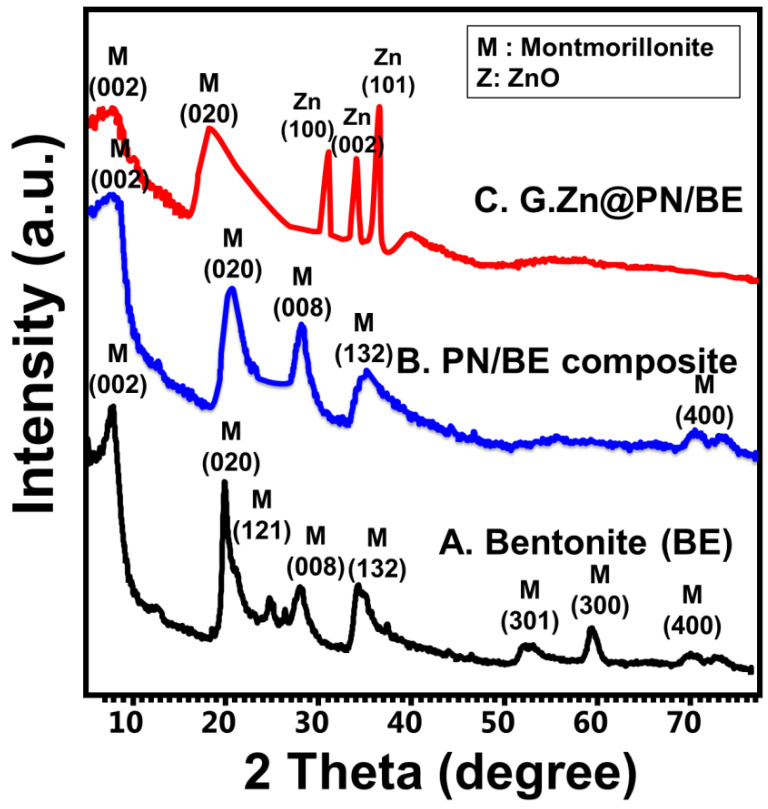
XRD patterns of raw bentonite (BE) (**A**), polyaniline/bentonite composite (PN/BE) (**B**), and the synthetic G.Zn@PN/BE green composite (**C**).

The change in the crystalline phases during the formation of the structure was assessed considering the XRD patterns. The bentonite substrate was confirmed by detecting numerous peaks related to the montmorillonite content (6.95°, 19.69°, and 25.12°) (XRD card No: 00-003-0010, 00-012-0232, and 00-058-2010) (Figure 1A). Comparing the PN/BE structure with bentonite and PANI, the observed pattern demonstrates fluctuation for the main montmorillonite peak to a high position (7.07°) (Figure 1B). This confirms the intercalation of the bentonite layer with PANI chains, which was reflected also in increasing the basal distance up to 13.64 Å instead of 12.7 Å (Figure 1B). The formation of G.Zn@PN/BE was confirmed by detecting the bentonite peak (7.12°) in addition to well-organized peaks for ZnO (31.7°, 34.5°, and 36.31°) with a crystallite size of 34 nm (JCPDS no. 65-3411; JCPDS no. 36-1451) (Figure 1C).

**Figure 2:**
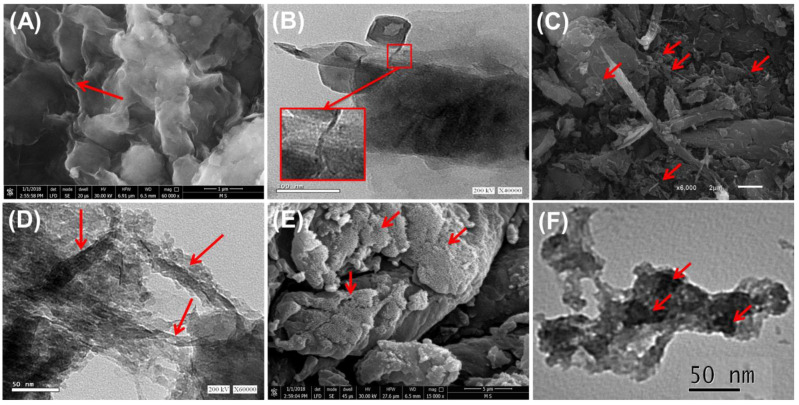
SEM image of raw bentonite (BE) (red arrows refer to the cornflakes structure) (**A**), HRTEM image of bentonite (**B**), SEM image of PN/BE composite (**C**) HRTEM image of PN/BE composite (red arrows refer to the polyaniline tubes) (**D**), SEM of G.Zn@PN/BE green composite (red arrows refer to the green ZnO particles) (**E**), and HRTEM image of G.Zn@PN/BE green composite (red arrows refer to the green ZnO particles) (**F**).

Comparing the morphological features of the combined component with the produced PN/BE and G.Zn@PN/BE structures confirm the successful integration between them (Figure 2). The SEM image of bentonite shows the characteristic form of the well-developed flexed platelets that interconnect with each other, creating the characteristic corn-flakes structure (Figure 2A). The TEM images reflect its multilayer structure, known as montmorillonite lattice fingers (Figure 2B). For the SEM images of the PN/BE structure, the PANI tubes were reported as needle-like or fibrous particles on the surface of the BE particles (Figure 2C). The TEM observations of PN/BE demonstrate remarkable destruction for the BE layers and orientation of the particles in a tabular form with numerous pores related to the interconnection between the tabular grains (Figure 2D). Regarding the synthetic G.Zn@PN/BE, the SEM image reflects the dispersion of the green ZnO grains that have a spherical morphology on the surface of PN/BE particles (Figure 2E). This was also detected in the TEM images, demonstrating the existence of ZnO as disseminated nanoparticles within the prepared PN/BE substrate (Figure 2F). Considering the effect of this on the microstructural properties, the determined surface area of G.Zn@/PN/BE is 145 m^2^/g, while the measured values of BE and PN/BE are 91 m^2^/g and 127 m^2^/g, respectively. The reported pore volumes are also enhanced significantly (BE (0.312 cm^3^/g), PN/BE (0.341 cm^3^/g), and G.Zn@PN/BE (0.403 cm^3^/g)) in addition to the average pore diameter (BE (10.4 nm), PN/BE (12.7 nm), and G.Zn@PN/BE (8.7 nm).

**Figure 3:**
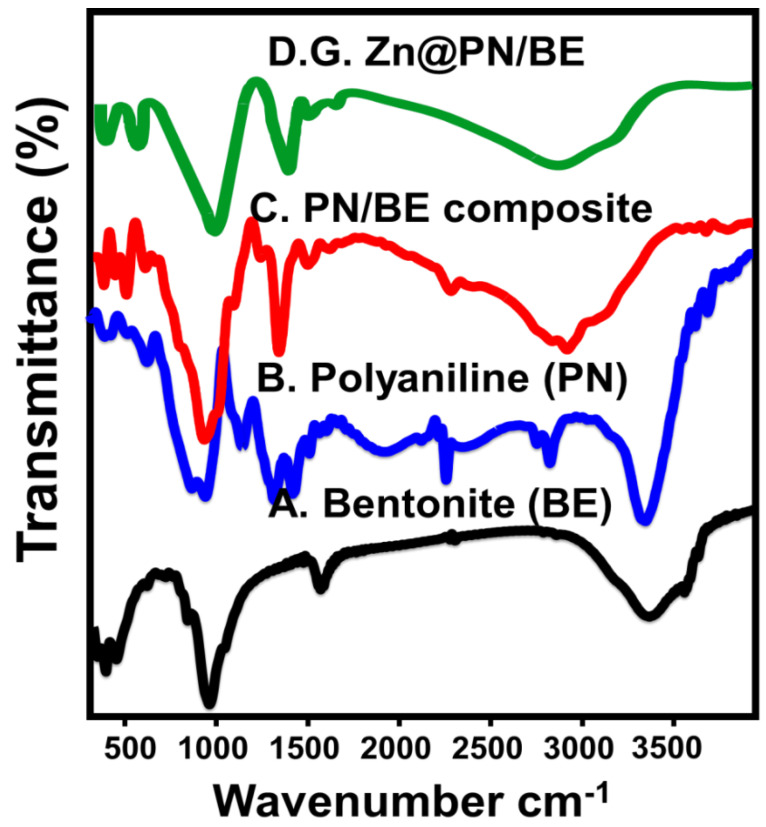
FT–IR spectra of raw bentonite (BE) (**A**), polyaniline (**B**), polyaniline/bentonite composite (PN/BE) (**C**), and the synthetic G.Zn@PN/BE green composite (**D**).

Such integration reactions also appeared in the identified chemical groups based on the FT-IR spectra (Figure 3). However, the bentonite and PANI samples show their identified chemical groups (Figure 3A,B), and the synthetic PN/BE structures exhibit mixing chemical groups related to both of them (Figure 3C). The bentonite groups are structural OH (3400 cm^−1^), interlayer water (1640 cm^−1^), Si–O (1000 cm^−1^), Al–O (918 cm^−1^), and Si–O–Al (400–1000 cm^−1^) (Figure 3A) [40]. The PANI groups are N-H (3401 cm^−1^), aromatic C–H (2918 cm^−1^), quinoid C=C (1467 cm^−1^), C=C of benzenoid (1301 cm^−1^), C–N (1105 cm^−1^), and C–H bending (789 cm^−1^) (Figure 3B) [25]. The identified groups of the PN/BE structure are OH, Si–O, and Al–O, which signifies the bentonite component (Figure 3C). The other identified groups are aromatic C–H, quinoid C=C, and benzenoid C=C, which signifies the PANI component (Figure 3C). Regarding the spectrum of G.Zn@PN/BE, it shows the same chemical groups of PN/BE in addition to two bands at about 530.6 cm^−1^ and 467.8 cm^−1^, signifying the integrated G.ZnO (O–Zn–O) (Figure 3D) [32]. This is in agreement with the findings of the EDX results, as the product is composed of O, Si, Al (bentonite component), C (PANI component), and Zn (G.ZnO component) (Figure S1). Therefore, the synthetic structure possesses multi-active chemical groups which have a strong impact on the retention of As (V) ions from water.

### 3.2. Retention Results

#### 3.2.1. Retention pH

**Figure 4:**
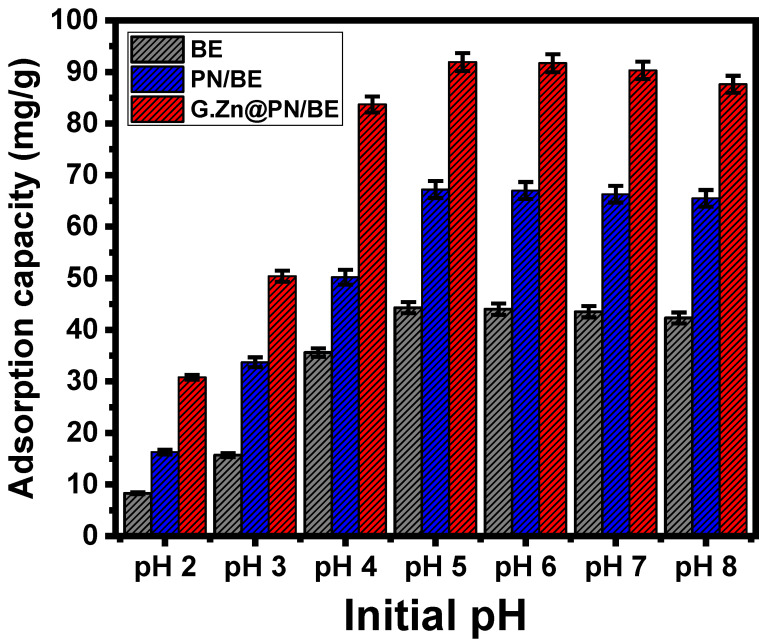
The influence of pH on the retention of As (V) ions by BE, PN/BE and G.Zn@PN/BE composite.

The pH as an experimental factor was assessed from pH 2 up to pH 8, considering the other variables at 200 mL as the volume, 100 mg/L as As (V) concentration, 120 min as retention interval, 0.1 g/L as the used dosages of adsorbents, and 20 °C as retention temperature. As can be concluded from the curves, BE, PN/BE, and G.Zn@PN/BE show enhancement in their As (V) uptake capacities with testing the high values of pH up to pH 5 (47.6 mg/g for BE, 58.2 mg/g for PN/BE, and 82.5 mg/g for G.Zn@PN/BE) (Figure 4). After that, the conducted experiments at pH values higher than pH 5 show considerable declination in the determined As (V) adsorption capacities until pH 8 for BE, in addition to its base structures (Figure 4). The speciation of As (V) at different pH values has a significant impact on the reported adsorption behaviors. The neutral form of As (V) (H_3_AsO_4_) can be detected from pH 2 up to pH 4 and the acidic forms as HAsO_4_^2−^ and AsO_4_^3−^ were recognized as dominant species from pH 7 up to pH 12 [8,41,42]. The acidic forms of As (V) at high pH conditions have remarkable repulsive properties with surficial chemical groups of BE, PN/BE, and G.Zn@PN/BE, which are affected by the de-protonation effect and are negatively charged and saturated by hydroxyl ions. Therefore, the adsorption of As (V) by BE, PN/BE, and G.Zn@PN/BE at pH 5 was recommended as it can keep significant quantities of positively charged groups on the surfaces of the adsorbents for the effective electrostatic attractions of the acidic forms of As (V) ions [43].

#### 3.2.2. Retention Time Interval

**Figure 5:**
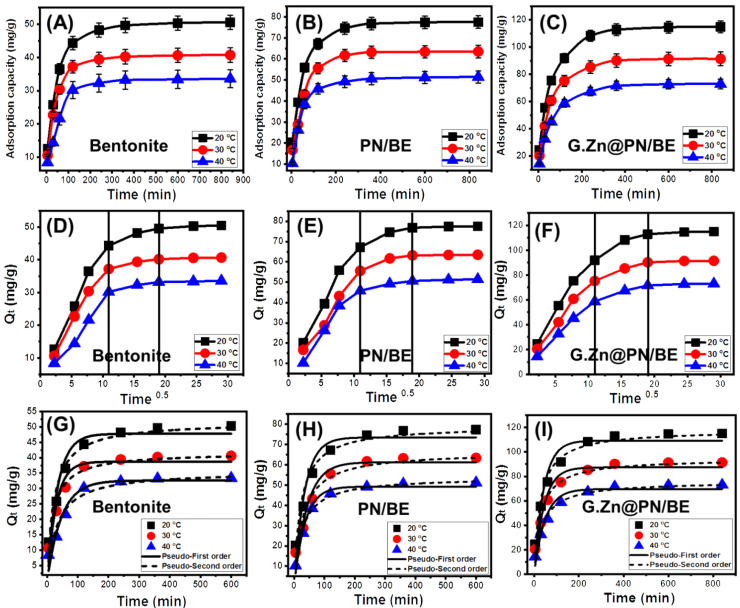
The retention behavior of As (V) ions by BE, PN/BE, and G.Zn@PN/BE as function of time (**A**–**C**), the Intra-Particle diffusion curves of As (V) adsorption results (**D**–**F**), and fitting of the As (V) retention results with the different kinetic models (**G**–**I**).

The As (V) retention behaviors of BE, PN/BE, and G.Zn@PN/BE with expanding the experimental interval were evaluated regularly up to 840 min at three retention temperature values (20 °C, 30 °C, and 40 °C). The main controlling variables were studied at 200 mL as volume, 100 mg/L as As (V) concentration, pH 5, and 0.1 g/L as the used dosages of adsorbents. The As (V) retention curves of BE, PN/BE, and G.Zn@PN/BE at the three values of temperature show the common segmental forms (Figure 5A–C). The rapid retention of As (V) can be observed by increasing the considered intervals up to 360 min for the G.Zn@PN/BE composite, in addition to its integrated components of bentonite (BE) and PN/BE composite. The previous interval represents the equilibrium point above which the actual retention of As (V) has slow rates and nearly fixed values. The equilibration was recognized as a result of the continuous occupation of BE, PN/BE, and G.Zn@PN/BE free effective sites with the adsorbed As (V) ions up to certain intervals after them there are-no or neglected remaining active sites (Figure 5A,C) [1].

The As (V) equilibration capacities of BE are 50.5 mg/g, 40.7 mg/g, and 33.6 mg/g at 20 °C, 30 °C, and 40 °C, respectively (Figure 5A). For PN/BE, the values increased to 77.5 mg/g (20 °C), 63.5 mg/g (30 °C), and 51.4 mg/g (40 °C) (Figure 5B), while the obtained values for the G.Zn@PN/BE composite are 115 mg/g (20 °C), 91.4 mg/g (30 °C), and 73 mg/g (40 °C) (Figure 5C). The enhancement in the As (V) retention capacity in the low-temperature conditions demonstrates the exothermic properties of the occurred reactions. Additionally, the reported declination in the equilibrium interval with decreasing temperature is related to the exothermic properties of the reactions, which causes complete occupation for the present sites with the As (V) at short intervals.

#### 3.2.3. Kinetic Studies

##### Intra-Particle Diffusion Behavior

The resulting intra-particle-diffusion curves for the occurred As (V) retention process using BE, PN/BE, and G.Zn@PN/BE adsorbents are presented in Figure 5D–F. The segmental shapes of the curves and the detection of no intersection with the original points suggest the significant effect of other adsorption mechanisms rather than the diffusion of As (V) ions [43] (Figure 5D–F). There are three observable segments in the curves, declaring three affecting mechanisms during the As (V) retention processes. The presence of surficial or external As (V) retention mechanisms was predicted from the first segment. The detection of the second segment suggests the vanishing of the effect of the external As (V) adsorption mechanism and the dominant impact on the layered adsorption mechanisms [44] (Figure 5D–F). During the equilibrium period, the third segment was detected, declaring the uptake of As (V) by interionic attraction presses and/or molecular association mechanisms. This is associated with the formation of a thick layer of the adsorbed As (V) ions on the surfaces of BE, PN/BE, and G.Zn@PN/BE [44].

##### Kinetic Modeling

The kinetic investigation of the As (V) adsorption processes using BE, PN/BE, and G.Zn@PN/BE was completed considering the nonlinear fitting mathematical parameters correlation coefficient (R2) and Chi-squared (χ^2^) with both Pseudo-First order (P.F) and Pseudo-Second order (P.S) models (Figure 5G–I). The values of both R^2^ and χ^2^ demonstrate higher agreement with the kinetic properties of the P.F model for G.Zn@PN/BE, as well as its integrated components (BE and PN/BE), at the three temperature values (20 °C, 30 °C, and 40 °C) (Figure 5G–I; Table 1). This suggested a dominant impact on physisorption mechanisms during the uptake of As (V) by the addressed structures [45,46]. However, the estimated significant fitting degrees with the assumption of the P.S model demonstrated a considerable effect on the chemisorption mechanisms that might be related strongly to the formation of chemical complexes, or the ion exchange processes with exchangeable ions of bentonite [19].

#### 3.2.4. As (V) Concentration

**Figure 6:**
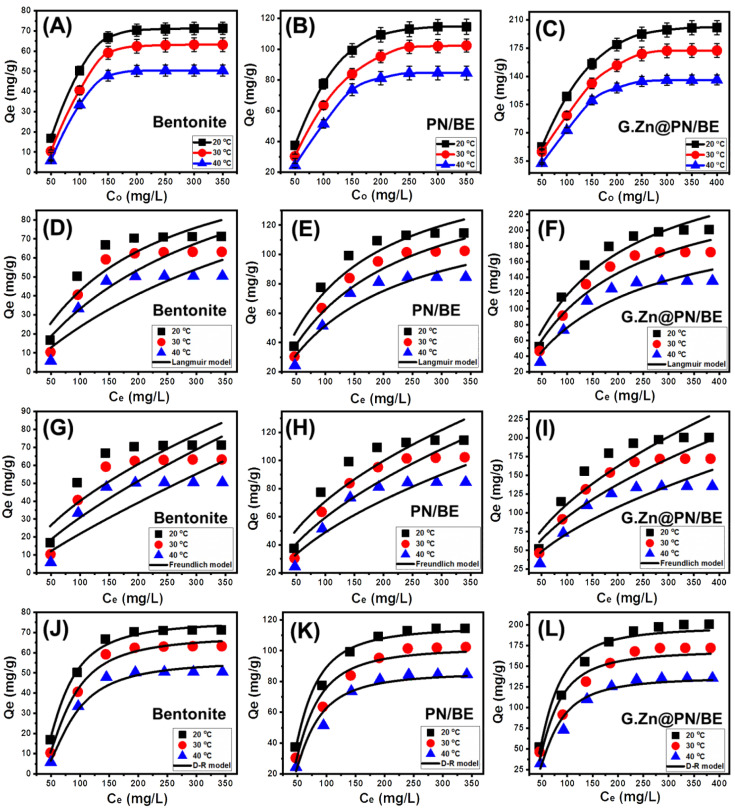
The retention behavior of As (V) ions by BE, PN/BE, and G.Zn@PN/BE as function of the initial concentration (**A**–**C**), fitting of the As (V) adsorption results with the Langmuir model (**D**–**F**), fitting of the As (V) retention results with the Freundlich model (**G**–**I**), and fitting of the As (V) adsorption results with the D-R model (**J**–**L**).

The influence of As (V) concentration on the actual BE, PN/BE, and G.Zn@PN/BE uptake capacities was evaluated from 50 mg/L up to 400 mg/L at three retention temperature values (20 °C, 30 °C, and 40 °C). The main controlling variables were studied at 200 mL as volume, 100 mg/L as As (V) concentration, pH 5, 840 min as time interval, and 0.1 g/L as the used dosages. The recognized As (V) retention capacities of BE, PN/BE, and G.Zn@PN/BE increased significantly when testing higher concentrations of the dissolved metal, especially in low-temperature conditions (Figure 6A–C). This was assigned to predict an increase in the As (V) driving forces as dissolved ions, which enhance the contact chances between the metal ions and the retentions sites of BE, PN/BE, and G.Zn@PN/BE [1]. Such an increment in the As (V) retention capacities was observed up to the testing concentrations of 200 mg/L for BE and 250 mg/L for PN/BE and G.Zn@PN/BE (Figure 6A–C). These As (V) concentrations are the equilibrium concentrations and beyond them, there are no remarkable changes in the determined retention capacities, demonstrating their maximum As (V) retention capacities. The determined actual maximum As (V) retention capacities of BE, PN/BE, and G.Zn@PN/BE at the best temperature (20 °C) are 71.3 mg/g, 114.6 mg/g, and 200.3 mg/g, respectively (Figure 6A–C). The measured As (V) retention capacity of BE enhanced significantly after intercalation of its layers with PANI (PN/BE) and after the decoration of the PN/BE structure with green ZnO particles. This reflected the positive effect of the integrated component, which might be related to the enhancement in the surface area and the incorporation of additional active retention sites.

#### 3.2.5. Classic Isotherm Models

The equilibrium properties of the As (V) adsorption processes using BE, PN/BE, and G.Zn@PN/BE were assessed based on the nonlinear fitting mathematical parameters correlation coefficient (R^2^) and Chi-squared (χ^2^) with Langmuir (L.G) (Figure 6D–F), Freundlich (F.E) (Figure 6G–I), and Dubinin–Radushkevich (D-R) (Figure 6J–L). Considering R^2^ as well as χ^2^ values, the retention processes of As (V) by BE, PN/BE, and G.Zn@PN/BE have Langmuir equilibrium properties rather than the Freundlich isotherm (Table 1). This suggests the possible retention of As (V) in monolayer form based on homogenous active receptors on the surface of G.Zn@PN/BE, in addition to its integrated component (BE and PN/BE) [47]. Based on the estimated mathematical parameters of the Langmuir isotherm, the theoretical maximum retention capacities (*Q*_max_) of BE, PN/BE, and G.Zn@PN/BE at 20 °C as the best temperature are 150.6 mg/g, 170.4 mg/g, and 311 mg/g, respectively (Table 1). The fitting degree with the Langmuir isotherm increases significantly after the integration of PANI in the PN/BE composite and the green ZnO in the synthetic G.Zn@PN/BE composite.

Considering the calculated mathematical parameters of the assessed D-R model, the values of Gaussian energy for BE, PN/BE, and G.Zn@PN/BE are within the range of physisorption processes (<8 kJ/mol) (Figure 6J–L; Table 1) [43]. There is a systematic declination in the values with the increase in As (V) retention temperature, reflecting the effective role of temperature in inducing the physical processes. Additionally, the determined values at the three temperatures (20 °C, 30 °C, and 40 °C) for G.Zn@PN/BE are lower than PN/BE and BE (G.Zn@PN/BE < PN/BE < BE), which demonstrates the impact of the integrated components in enhancing the efficiency of the physical mechanisms during the retention of As (V).

#### 3.2.6. Advanced Isotherm Models

**Figure 7:**
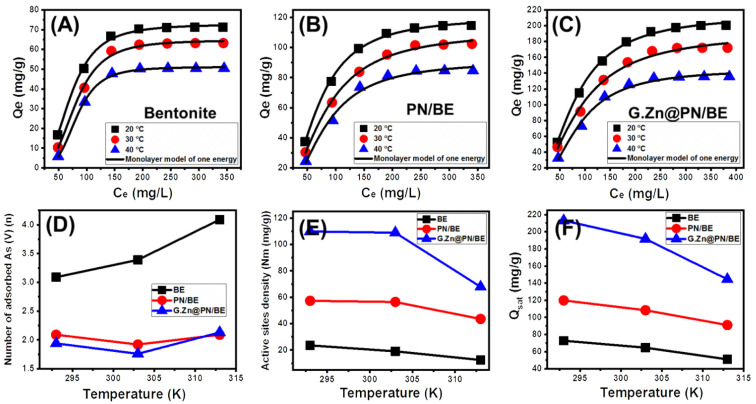
Fitting of the As (V) retention results by BE, PN/BE, and G.Zn@PN/BE with advanced monolayer model of one energy (**A**–**C**), the behavior of *n* parameter (number of adsorbed As (V) ions per site) at different temperature values (**D**), the behavior of Nm parameter (active sites density) at different temperature values (**E**), and the behavior of Q_sat_ parameter (adsorption capacity at saturation) at different temperature values (**F**).

Considering the values of the correlation coefficient (R^2^) and the root mean square error (RMSE), the retention of As (V) by BE, PN/BE, and G.Zn@/PN/BE can be assessed based on the assumption of the monolayer model with one energy (Model.1) compared to the other studied advanced models (Figure 7A–C). The mathematical parameters of the model were used to illustrate the steric (*n* (the number of adsorbed As (V) ions), Nm (the occupied receptor sites), and Q_sat_ (the adsorption capacity as the saturation state)) and energetic properties (∆E (As (V) adsorption energy), S_a_ (entropy), G (enthalpy), and E_int_ (internal energy)) of the As (V) retention systems (Table 2). Such parameters are significant indicators of the effect of the composite components on the retention efficiency of the As (V) ions.

##### Steric Properties

Number of adsorbed As (V) ions per site (*n*)

The *n* parameter (adsorbed As (V) ions per site) strongly indicates the retention mechanism of As (V) ions and the orientation of the adsorbed As (V) ions on the surfaces of BE, PN/BE, and G.Zn@PN/BE. All the estimated *n* values for the retention of As (V) by G.Zn@PN/BE and its components (E and PN/BE) are higher than 1 (Figure 7D; Table 2). This suggested that the retention of As (V) ions were of non-parallel and/or vertical orientation on the surfaces of the adsorbents. Additionally, this signifies the retention of the dissolved As (V) ions on the adsorption sites by a multi-ionic mechanism [48,49]. The determined *n* values for BE within the range from 3.09 up to 4.09 suggest the uptake of three or four As (V) ions per site on the surface of BE particles (Figure 7B; Table 2). These values are higher than the estimated values for PN/BE (1.92 up to 2.09) and the synthetic G.Zn@PN/BE (1.76 up to 2.13) (Figure 7D; Table 2). This might be related to the increase in the number of active adsorption sites after the integration of PANI chains and the green ZnO particles.

Regarding the impact of temperature, the *n* values declined regularly with the increase in temperature during the retention of As (V) by BE particles. This might be related to the impact of temperature in the activation of new sites or the ion exchange process, causing a reduction in the number of captured As (V) per site. For PN/BE and G.Zn@PN/BE, the *n* values declined with temperature up to 30 °C and then increased again up to 40 °C. This was reported in the literature as a predicted result for the change in the effective retention sites with the temperature [49,50].

Density of the active sites (Nm)

The values of the Nm parameter (density of adsorption sites) during the retention of As (V) by G.Zn@PN/BE are higher than the values of PN/BE and BE (G.Zn@PN/BE > PN/BE > BE) (Figure 7E; Table 2). This declares the effect of the integrated components (PNAI (PN) and green ZnO (G.Zn)) in inducing the quantities of the active sites on the surface of the final hybrid structure by providing it with new adsorption sites. Regarding the effect of temperature, there is a considerable declination in the calculated values of the Nm parameter for BE with the increase in experimental temperature from 20 °C up to 40 °C (Figure 7E; Table 2). The previous behavior was credited to the increase in the aggregation of the adsorbed As (V) ions, which appeared significantly in the values of the *n* parameter [49]. The enhancement in the aggregation properties (*n* parameter) is associated with declination in the number of the occupied active sites. Moreover, the aggregation process causes a decrease in the interaction chances between As (V) ions on the present free active adsorption sites.

The saturation As (V) adsorption capacity (Q_sat_)

The As (V) adsorption capacity at the saturation state (Q_sat_) is controlled essentially by the *n* parameter (number of adsorbed As (V) ions) and/or Nm parameter (density of active sites). There is considerable declination in the estimated Q_sat_ values with the increase in test temperature from 20 °C (BE (72.7 mg/g), PN/BE (119.8 mg/g), and G.Zn@PN/BE (213 mg/g)) up to 40 °C (BE (51 mg/g), PN/BE (90.9 mg/g), and G.Zn@PN/BE (144.5 mg/g)) (Figure 7F; Table 2). This behavior is in agreement with the reported behavior for the values of the Nm parameter (density of adsorption sites). Therefore, the As (V) adsorption capacities of G.Zn@PN/BE and its components (BE and PN/BE) are controlled by the availability and quantities of the present active adsorption sites. This also illustrates the enhancement effect of the integrated components, as they provided the structure with new and more active sites.

##### Energetic Properties

Adsorption energy

The adsorption energy values of the As (V) retention reactions by BE, PN/BE, and G.Zn@PN/BE were calculated according to Equation (5) [49].

(5) $$∆E=RT\;ln\left(\frac{S}{C}\right)$$

The presented symbols in the formula denote the adsorption energ (∆E), gas constant (*R*), absolute temperature (*T*), the solubility of As (V) (*S*), and As (V) concentration during the half-saturation state (*C*). The values of the adsorption energies reflect strongly the nature of the controlling mechanisms during the retention of As (V) ions. Adsorption systems with energy lower than 40 kJ/mol are controlled by physical mechanisms such as hydrogen bonding (<30 kJ/mol), van der Waals forces (4–10 kJ/mol), hydrophobic bonding (5 kJ/mol), coordination exchange process (40 kJ/mol), and dipole bonding forces (2–29 kJ/mol) [49,51]. The determined values for the retention of As (V) by BE, PN/BE, and G.Zn@PN/BE at the tested temperature values are higher than 3 kJ/mol and lower than 4 kJ/mol (Table 2). These values reflect the controlling effect for the physical mechanisms during the As (V) uptake reactions (hydrogen bonding and dipole bonding forces). The estimation of As (V) adsorption energies as negative values demonstrates the exothermic properties of the occurred retention reactions by BE, PN/BE, and G.Zn@PN/BE [52].

Thermodynamic functions
  Internal energy and free enthalpy

The internal energy of As (V) retention reactions by G.Zn@PN/BE and its components (BE and PN/BE) was calculated considering Equation (6), in which the Zv symbol refers to the translation partition value per unit volume [52].

(6) $$\frac{E_{\mathrm{int}}}{K_{B}T\;}=n\;N_{m}\left[\left(\;\frac{{\left(\frac{C}{C_{1/2}}\right)}^{n}\;ln\left(\frac{C}{Z_{v}}\right)}{1+{\left(\frac{C}{C_{1/2}}\right)}^{n}}\right)-\left(\;\frac{n\;ln\left(\frac{C}{C_{1/2}}\right)\;{\left(\frac{C}{C_{1/2}}\right)}^{n}}{1+{\left(\frac{C}{C_{1/2}}\right)}^{n}}\right)\right]$$

**Figure 8:**
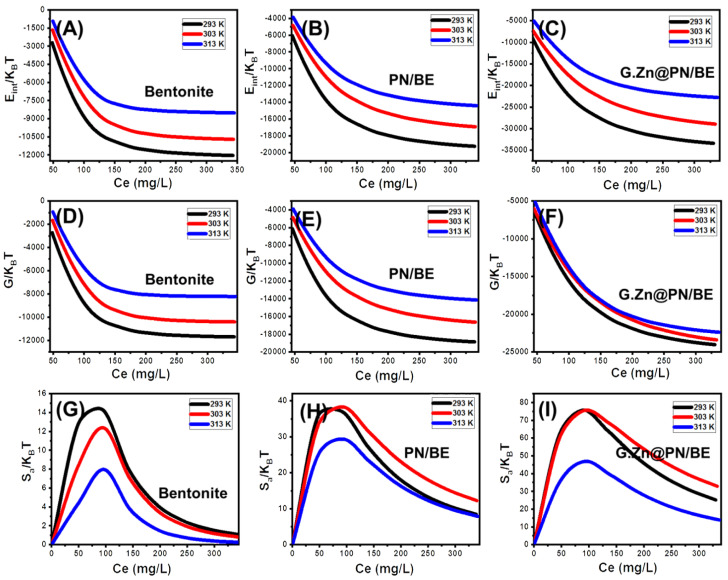
The behavior of the internal energy (E_int_) during the retention processes at different concentrations of As (V) ions (**A**–**C**), the behavior of the free enthalpy during the retention processes at different concentrations of As (V) ions (**D**–**F**), and the behavior of the entropy (S_a_) during the retention processes at different concentrations of As (V) ions (**G**–**I**).

The obtained internal energies (E_int_) for the retention of As (V) by BE, PN/BE, and G.Zn@PN/BE are negative signs (Figure 8A–C). This reflects the spontaneous properties of the occurred retention reactions at the investigated As (V) concentrations and temperature values. The observed declination in the E_int_ values with temperature from 20 °C up to 40 °C demonstrates the exothermic properties of the As (V) retention reactions (Figure 8A–C). This was supported by the inspected free enthalpy (G) values of the reactions based on Equation (7) [52]. The calculated free enthalpy values are also negative signs at all the addressed As (V) concentrations and temperature values (Figure 8D–F). This confirms the previous findings from the E_int_ values about the spontaneous properties of the As (V) retention reactions by G.Zn@PN/BE and its components (BE and PN/BE). The reported enhancement in the G values with the decrease in experimental temperature reflects an enhancement in the feasibility of the As (V) retention reactions in low temperature conditions (Figure 8D–F).

(7) $$\frac{G}{K_{B}T\;}=n\;N_{m}\frac{ln\left(\frac{C}{Z_{v}}\right)}{1+{\left(\frac{C_{1/2}}{C}\right)}^{n}}$$

Entropy

The entropy of the As (V) retention system (S_a_) considering both the metal concentration and retention temperature can signify the order and disorder properties of the surfaces of G.Zn@PN/BE and its components (BE and PN/BE) as adsorbents. The values of entropy were calculated according to Equation (8) [52].

(8) $$\frac{S_{a}}{K_{B}}=\mathrm{Nm}\left\{ln\left(1+{\left(\frac{C}{C_{\frac{1}{2}}}\right)}^{n}\right)-n{\left(\frac{C}{C_{\frac{1}{2}}}\right)}^{n}\;\frac{ln\left(\frac{C}{C_{\frac{1}{2}}}\right)}{1+{\left(\frac{C}{C_{\frac{1}{2}}}\right)}^{n\;}}\;\;\right\}$$

The determined entropy values show reversible trends with the addressed As (V) concentrations reflecting a significant increase in the disorder properties of BE, PN/BE, and G.Zn@PN/BE during the retention of the metal ions (Figure 8G–I). This demonstrates the docking of the As (V) ions on the present active receptors of the studied structures with conducting the tests at low metal concentrations [49,50]. For BE, the maximum values of entropy were detected at As (V) equilibration concentrations of 88.5 mg/L (293 K), 90.8 mg/L (303 K), and 92.7 mg/L (313 K) (Figure 8G). For PN/BE, the maxima values were identified at the concentrations of 94.9 mg/L (293 K), 95.9 mg/L (303 K), and 96.6 mg/L (313 K) (Figure 8H), while the identified concentrations for G.Zn@PN/BE are 92.2 mg/L (293 K), 93.6 mg/L (303 K), and 94.8 mg/L (313 K) (Figure 8I). These values are close to the obtained values for the concentrations of As (V) at half-saturation ($C_{1/2}$). This suggests the reorganization of the saturation values of the present active retention sites of the products and no additional As (V) ions can be docked on them following this. Beyond these concentrations, the determined S_a_ values declined significantly, signifying the reduction in the available active sites, the freedom degrees, and diffusion properties of the As (V) ions [48].

#### 3.2.7. Effect of Coexisting Cations and Anions

**Figure 9:**
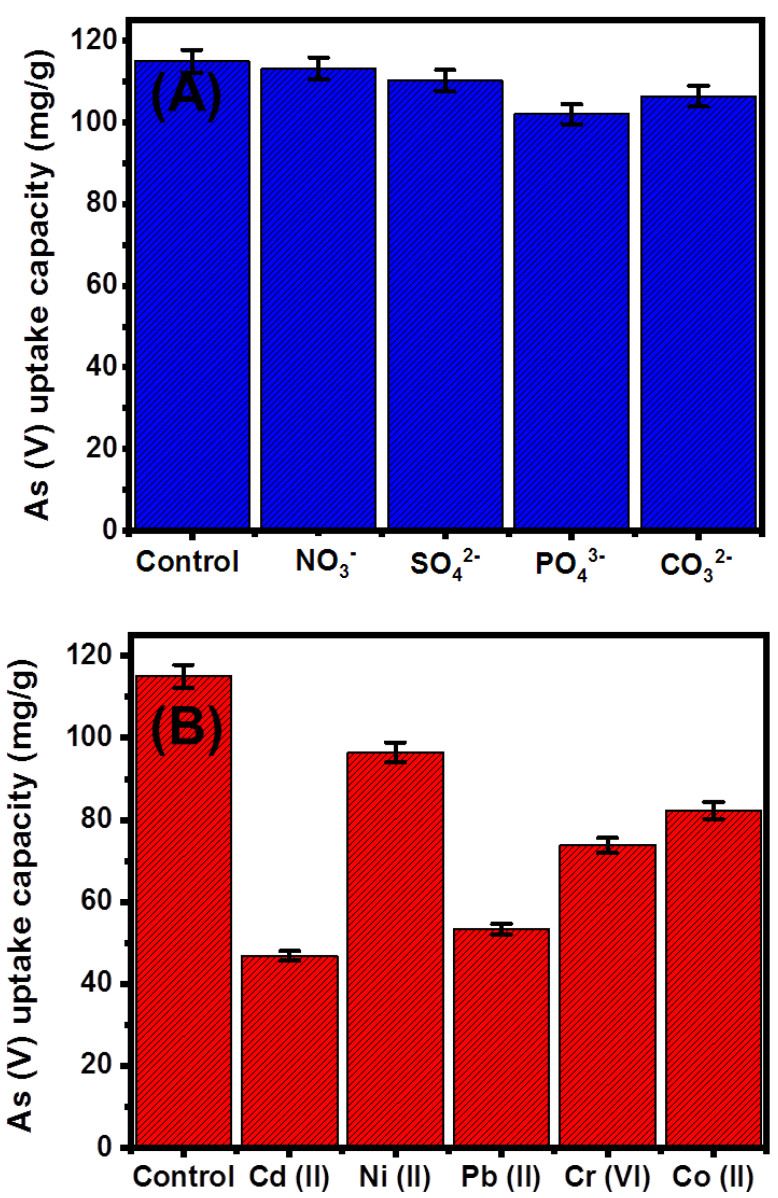
Effect of the competitive chemical anions (**A**) and metal ions (**B**) on the retention capacity of As (V) ions by G.Zn@PN/BE.

The influence of the other coexisting cations (Cd (II), Ni (II), Pb (II), Cr (VI), and Co (II)), as well as the anions (NO_3_^−^, SO_4_^2−^, PO_4_^3−^, and CO_3_^2−^), on the efficiency and selectivity of G.Zn@PN/BE as adsorbent for As (V) ions was evaluated experimentally. The main controlling variables were studied at 200 mL as volume, 100 mg/L as tested concentration (50% As (V) + 50% coexisting ions), pH 5, and 0.1 g/L as G.Zn@PN/BE dosage, 20 °C as retention temperature, and 840 min as a time interval (Figure 9).

The addressed anions (NO_3_^−^, SO_4_^2−^, PO_4_^3−^, and CO_3_^2−^) have a low negative effect on the retention efficiency of As (V) by G.Zn@PN/BE (Figure 9A). Both PO_4_^3−^ and CO_3_^2−^ have a higher competitive effect during the retention of As (V) compared to NO_3_^−^ and SO_4_^2−^ ions (Figure 9A). This was reported in the literature as a result of the significant similarity between As (V) and PO_4_^3−^ ions in their chemical behaviors and physicochemical properties [53]. Additionally, the retention of PO_4_^3−^ ions occurred by inner-sphere complexes with the OH-bearing functional groups which induce their competitive effect with the As (V) ions [41]. This was also reported for the retention of CO_3_^2−^ as competitive ions for As (V) metal, as both have similar molecular structures [41]. The retention of NO_3_^−^ and SO_4_^−^ ions occurred by outer-sphere complexes, which make them of low competitive impacts during the retention of As (V) by G.Zn@PN/BE, which involved the formation of stable inner-sphere complexes [41,54].

Regarding the competitive impact of the other metals (Cd (II), Ni (II), Pb (II), Cr (VI), and Co (II)) during the retention of As (V), they have a remarkable negative influence on the efficiency of G.Zn@PN/BE as an adsorbent (Figure 9B). The presence of Cd (II), Ni (II), Pb (II), Cr (VI), and Co (II) ions reduce the efficiency of G.Zn@PN/BE during the retention of As (V) to 46.8 mg/g, 96.5 mg/g, 53.3 mg/g, 73.8 mg/g, and 82.3 mg/g, respectively (Figure 9B). However, the presence of these dissolved metals has a significant adverse and competitive impact during the retention of As (V) ions; the determined results considering the tested concentration qualify the synthetic structure to be applied in realistic remediation processes.

#### 3.2.8. Recyclability

The reusability properties of the synthetic G.Zn@PN/BE structure were assessed after the alkaline washing step (20 mL; 0.05 M NaOH) at 20 °C for 120 min. Then, the composite particles were washed with distilled water until neutralization and dried at 60 °C for 8 h. This was repeated after each adsorption cycle considering the total cycles at 5 runs (Figure S2). The As (V) retention recyclability tests were performed at experimental conditions of 200 mL as volume, 100 mg/L as tested As (V) concentration, pH 5, and 0.1 g/L as G.Zn@PN/BE dosage, 20 °C as retention temperature, and 840 min as a time interval. The synthetic G.Zn@PN/BE structure as adsorbent for As (V) ions is of significant reusability value considering the estimated concentration and recyclability runs (Figure S2). The determined As (V) retention capacities at the five cycles are 115 mg/g (Cycle 1), 112 mg/g (Cycle 2), 107.6 mg/g (Cycle 3), 98.3 mg/g (Cycle 4), and 90.4 (Cycle 5) (Figure S2). The slight declination in the retention capacity with the increase in the number of the reusability cycles might be attributed to the increase in the number of formed complexes between the adsorbed As (V) metals and the effective chemical groups.

#### 3.2.9. Comparison Study

The As (V) retention results of the synthetic G.Zn@PN/BE were compared with other studied adsorbents. The structure exhibits higher As (V) retention capacity (213 mg/g) than its components of bentonite (BE) (72.7 mg/g), green synthesized ZnO (42.3 mg/g), and PNAI/bentonite composite (PN/BE) (119.8 mg/g). This signifies the impact of the combination process in enhancing the surface area and increasing the quantities of the active adsorption sites during the retention process. Moreover, the structure achieved higher efficiency than several studied adsorbents, as presented in Table 3. This declares the value of the structure, as it is of facile synthesis procedures and environmental properties. Therefore, the synthetic G.Zn@PN/BE tripartite hybrid structure can be applied effectively in the realistic removal of As (V) metal ions from different polluted water resources.

## 4. Conclusions

The adsorption properties of a green ZnO@polynaniline/bentonite composite (G.Zn@PN/BE) for As (V) ions were evaluated considering the effect of its components (BE and PN/BE). The G.Zn@PN/BE composite showed significant efficiency (213 mg/g) compared to its components (BE (72.7 mg/g), ZnO (42.3 mg/g), and PN/BE (119.8 mg/g)) or other studied absorbents. The adsorption behavior and mechanism were assessed based on the assumptions of the classic Langmuir and advanced monolayer model, one of the energy models. Besides the reported enhancement in the surface area (145 m^2^/g), the steric parameter declared an increase in the quantities of the active adsorption sites after the integration process (Nm = 109.8 (20 °C), 108.9 (30 °C), and 67.8 mg/g (40 °C)). This illustrates the detected higher As (V) retention capacity by G.Zn@PN/BE than BE and PN/BE. Moreover, the values of *n* parameter demonstrate the retention of As (V) as two or three ions per site by a multi-ionic mechanism. Considering the values of adsorption energy (from −3.07 to −3.26 kJ/mol), the uptake of As (V) by G.Zn@PN/BE occurred by physisorption processes. The thermodynamic functions of internal energy, free enthalpy, and entropy declared the retention of As (V) by exothermic, feasible, and spontaneous reactions.

## Figures and Tables

**Table 1:** The mathematical parameters of the addressed kinetic and classic isotherm models.

Kinetic Models					
Material	Model	Parameters	293 K	303 K	313 K
**BE**	**Pseudo-First-order**	**K_1_ (1/min)**	0.0294	0.0335	0.0215
		***Qe*_(*Cal*)_ (mg/g)**	47.7	38.7	32.58
		**R^2^**	0.99	0.98	0.98
		**X^2^**	0.288	0.162	0.478
	**Pseudo-Second-order**	**k_2_ (mg/g min)**	8.71 × 10^−4^	0.0012	8.76 × 10^−4^
		***Qe*** **_(*Cal*)_ (mg/g)**	51.59	41.67	35.58
		**R^2^**	0.96	0.97	0.92
		**X^2^**	0.67	0.53	0.67
**PN/BE**	**Pseudo-First-order**	**K_1_ (1/min)**	0.029	0.023	0.028
		***Qe*** **_(*Cal*)_ (mg/g)**	73.5	61.29	49.42
		**R^2^**	0.99	0.99	0.99
		**X^2^**	1.55	0.79	0.115
	**Pseudo-Second-order**	**k_2_ (mg/g min)**	5.75 × 10^−4^	5.41 × 10^−4^	7.2 × 10^−4^
		***Qe*** **_(*Cal*)_ (mg/g)**	79.26	66.42	53.9
		**R^2^**	0.95	0.94	0.99
		**X^2^**	1.76	1.19	0.353
**G.Zn@PN/BE**	**Pseudo-First-order**	**K_1_ (1/min)**	0.0234	0.0229	0.020
		***Qe*** **_(*Cal*)_ (mg/g)**	109.2	87.4	69.67
		**R^2^**	0.99	0.99	0.99
		**X^2^**	0.515	0.53	0.34
	**Pseudo-Second-order**	**k_2_ (mg/g min)**	2.96 × 10^−4^	3.6 × 10^−4^	3.87 × 10^−4^
		***Qe*** **_(*Cal*)_ (mg/g)**	118.05	94.3	75.92
		**R^2^**	0.97	0.97	0.97
		**X^2^**	1.31	1.08	0.78
**Isotderm Models**					
**BE**	**Langmuir model**	***Q*_max_ (mg/g)**	150.06	140.6	124.2
		**b (L/mg)**	0.0018	0.003	0.0052
		**R^2^**	0.84	0.87	0.88
		**X^2^**	3.06	3.09	2.12
	**Freundlich model**	**1/*n***	0.595	0.728	0.843
		**k_F_ (mg/g)**	2.58	1.075	0.449
		**R^2^**	0.78	0.79	0.77
		**X^2^**	3.46	4.15	4.79
	**D-R model**	**β (mol^2^/KJ^2^)**	0.037	0.041	0.056
		***Q_m_* (mg/g)**	75.6	68.3	55.9
		**R^2^**	0.996	0.994	0.991
		**X^2^**	0.057	0.109	0.182
		**E (KJ/mol)**	3.83	3.49	2.98
**PN/BE**	**Langmuir model**	***Q*** **_max_ (mg/g)**	170.4	163.7	138.9
		**b (L/mg)**	0.0078	0.0061	0.0058
		**R^2^**	0.94	0.95	0.94
		**X^2^**	0.92	0.72	0.92
	**Freundlich model**	**1/*n***	0.49	0.54	0.55
		**k_F_ (mg/g)**	7.41	4.83	3.78
		**R^2^**	0.88	0.89	0.88
		**X^2^**	2.1	1.68	1.84
	**D-R model**	**β (mol^2^/KJ^2^)**	1.05	1.19	1.28
		***Q_m_* (mg/g)**	115.5	101.9	85.8
		**R^2^**	0.98	0.97	0.97
		**X^2^**	0.22	0.47	0.43
		**E (KJ/mol)**	0.69	0.64	0.62
**G.Zn@PN/BE**	**Langmuir model**	***Q*** **_max_ (mg/g)**	311.8	274	226.39
		**b (L/mg)**	0.006	0.0056	0.005
		**R^2^**	0.95	0.95	0.93
		**X^2^**	1.57	1.13	1.79
	**Freundlich model**	**1/*n***	0.54	0.54	0.57
		**k_F_ (mg/g)**	9.35	7.59	5.06
		**R^2^**	0.88	0.89	0.86
		**X^2^**	3.7	2.79	3.48
	**D-R model**	**β (mol^2^/KJ^2^)**	1.22	1.29	1.48
		***Q_m_* (mg/g)**	197.2	168.4	136.4
		**R^2^**	0.96	0.93	0.96
		**X^2^**	1.05	1.78	1.90
		**E (KJ/mol)**	0.64	0.62	0.58

**Table 2:** The estimated mathematical parameters for the fitting process with Monolayer model of one energy.

Steric and Energetic Parameters						
		*n*	Nm (mg/g)	Q_sat_ (mg/g)	*C*_1/2_ (mg/L)	∆E (kJ/mol)
**BE**	**293 K**	3.09	23.53	72.7	71.57	−3.50
	**303 K**	3.39	19.06	64.6	80.23	−3.33
	**313 K**	4.09	12.47	51	81.92	−3.39
**PN/BE**	**293 K**	2.09	57.33	119.8	67.94	−3.63
	**303 K**	1.92	56.43	108.3	78.38	−3.39
	**313 K**	2.09	43.54	90.99	78.35	−3.51
**G.Zn@PN/BE**	**293 K**	1.94	109.86	213.1	80.52	−3.22
	**303 K**	1.76	108.9	191.6	89.23	−3.07
	**313 K**	2.13	67.88	144.5	86.13	−3.26

**Table 3:** Comparison between the As (V) retention capacity of G.Zn@PN/BE composite and other studied adsorbents.

Adsorbent	*Q*_max_ (mg/g)	Reference
**Goetdite/goetdite P**	34.12	[16]
**ZnO/AlSBA-15**	123.99	[13]
**ZrO(OH)_2_/CNTs**	124.5	[55]
**0.26γ-Fe_2_O_3_/SBA-15**	23.09	[56]
**MWCNTs OCH_2_CO_2_H**	250	[14]
**FeOx-GO**	113	[57]
**Chitosan-coated biosorbent**	96.46	[58]
**Fe-MWCNTs**	250	[59]
**GONRs**	155.6	[60]
**MWCNTs**	200	[14]
**Iron oxide nanoparticle**	22.91	[61]
**Zirconium-nanoscale carbon**	110	[62]
**Silica-sand/cationized-starch**	76.63	[63]
**GO/CuFe_2_O_4_ foam**	124.69	[64]
**Green ZnO**	42.3	This study
**BE**	72.7	This study
**PN/BE**	119.8	This study
**G.Zn@PN/BE composite**	213	This study

## Data Availability

The data presented in this study are available on request from the corresponding author.

## Supplementary Materials

The following supporting information can be downloaded at: https://www.mdpi.com/article/10.3390/polym14122329/s1, Figure S1: EDX spectrum of the synthetic G.Zn@PN/BE green composite; Figure S2: Recyclability of G.Zn@PN/BE during the retention of As (V) ions from water; Table S1. Nonlinear equations of kinetic, classic isotherm, and advanced isotherm models.
