# Supplementary material for: Synthesis and Characterization of Green ZnO@polynaniline/Bentonite Tripartite Structure (G.Zn@PN/BE) as Adsorbent for As (V) Ions: Integration, Steric, and Energetic Properties
Source: Polymers (Basel). 2022 Jun 9;14(12):2329. doi: 10.3390/polym14122329 (PMC9229974; doi:10.3390/polym14122329)
Supplement: Supplementary file 1 [file polymers-14-02329-s001.zip › polymers-1756322-supplementary.pdf]

Synthesis and Characterization of Green ZnO@polynaniline/Bentonite Tripartite Structure (G.Zn@PN/BE) as Adsorbent for As (V) Ions: Integration, Steric, and Energetic Properties

Mohamed Abdel Salam [1], Mohamed Mokhtar [1], Soha M. Albukhari [1], Doaa F. Baamer [1], Leonardo Palmisano [2], Mariusz Jaremko [3] and Mostafa R. Abukhadra [4,5,*]

[1] Department of Chemistry, Faculty of Science, King Abdulaziz University, P.O. Box 80200, Jeddah 21589, Saudi Arabia; mabdelsalam@kau.edu.sa (M.A.S.); mmoustafa@kau.edu.sa (M.M.); salbukhari@kau.edu.sa (S.M.A.); dfbaamer@kau.edu.sa (D.F.B.)

[2] Schiavello-Grillone Photocatalysis Group, Dipartimento di Ingegneria, Università degli Studi di Palermo, Viale delle Scienze (Ed. 6), 90128 Palermo, Italy; leonardo.palmisano@unuipa.it

[3] Smart-Health Initiative (SHI), Red Sea Research Center (RSRC), Biological and Environmental Science and Engineering (BESE) Division, King Abdullah University of Science and Technology (KAUST), P.O. Box 4700, Thuwal 23955-6900, Saudi Arabia; mariusz.jaremko@kaust.edu.sa

[4] Geology Department, Faculty of Science, Beni-Suef University, Beni-Suef City 62511, Egypt

[5] Materials Technologies and Their Applications Lab, Geology Department, Faculty of Science, Beni-Suef University, Beni-Suef City 62111, Egypt

* Correspondence: abukhadra89@science.bsu.edu.eg

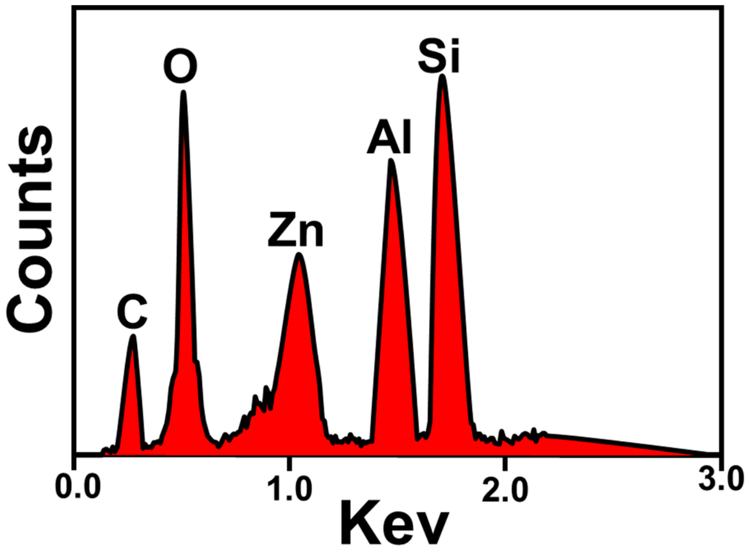

Figure S1. EDX spectrum of the synthetic G.Zn@PN/BE green composite.

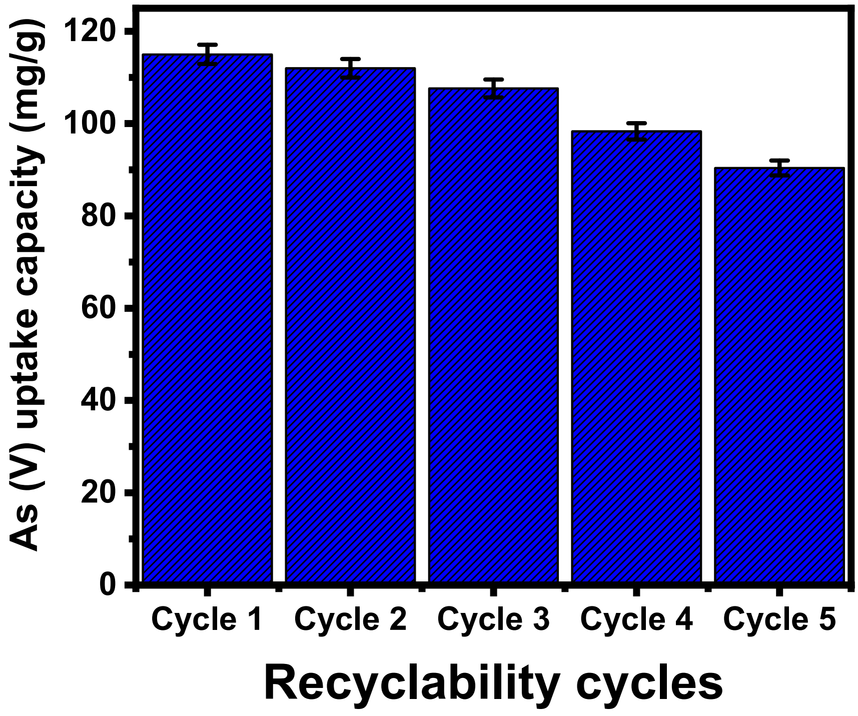

Figure S2. Recyclability of G.Zn@PN/BE during the retention of As (V) ions from water.

Table S1. Nonlinear equations of kinetic, classic isotherm, and advanced isotherm models.

	**Kinetic models**	
**Model**	**Equation**	**Parameters**
**Pseudo-first-order**	$Q_t = Q_e\,(1 - e^{-k_1.t})$	Q_t (mg/g) is the adsorbed ions at time (t), and K_1 is the rate constant of the first-order adsorption (1/min)
**Pseudo-second-order**	$Q_t = \frac{Q_e^2 k_2 t}{1 + Q_e k_2 t}$	Qe is the quantity of adsorbed ions after equilibration (mg/g), and K_2 is the model rate constant (g/mg min).
	**Classic Isotherm models**	
**Model**	**Equation**	**Parameters**
**Langmuir**	$Q_e = \frac{Q_{max}\, bC_e}{(1 + bC_e)}$	C_e is the rest ions concentrations (mg/L), Q_{max} is the theoritical maximum adsorption capacity (mg/g), and *b* is the Langmuir constant (L/mg)
**Freundlich**	$Q_e = K_f C_e^{1/n}$	K_F (mg/g) is the constant of the Freundlich model related to the adsorption capacity and n is the constant of the Freundlich model related to the adsorption intensities
**Dubinin–Radushkevich**	$Q_e = Q_m e^{-\beta\varepsilon^2}$	β (mol^2/KJ^2) is the D-R constant, ε (KJ^2/mol^2) is the polanyil potential, and Q_m is the adsorption capacity (mg/g)
	**Advanced isotherm models**	
**Model**	**Equation**	**Parameters**
**Monolayer model with one energy site (Model 1)**	$Q = nN_o = \frac{nN_M}{1 + (\frac{C1/2}{C})^n} = \frac{Q_o}{1 + (\frac{C1/2}{C})^n}$	Q is the adsorbed quantities in mg/g<br>n is the number of adsorbed ions per site
**Monolayer model with two energy sites (Model 2)**	$Q = \frac{n_1 N_{1M}}{1 + (\frac{C_1}{C})^{n_1}} + \frac{n_2 N_{2M}}{1 + (\frac{C_2}{C})^{n_2}}$	Nm is the density of the effective receptor sites (mg/g)<br>Q_o is the adsorption capacity at the saturation state in mg/g
**Double layer model with one energy site (Model 3)**	$Q = Q_o \frac{(\frac{C}{C1/2})^n + 2(\frac{C}{C1/2})^{2n}}{1 + (\frac{C}{C1/2})^n + (\frac{C}{C1/2})^{2n}}$	C1/2 is the concentration of the ions at half saturation stage in mg/L<br>C1 and C2 are the concentrations of the ions at the half saturation stage for the first active sites and the second active sites, respectively
**Double layer model with two energy sites (Model 3)**	$Q = Q_o \frac{(\frac{C}{C1})^n + 2(\frac{C}{C2})^{2n}}{1 + (\frac{C}{C1})^n + (\frac{C}{C2})^{2n}}$	n1 and n2 are the adsorbed ions per site for the first active sites and the second active sites, respectively
